# Genetic diversity of *SAD* and *FAD* genes responsible for the fatty acid composition in flax cultivars and lines

**DOI:** 10.1186/s12870-020-02499-w

**Published:** 2020-10-14

**Authors:** Alexey A. Dmitriev, Parfait Kezimana, Tatiana A. Rozhmina, Alexander A. Zhuchenko, Liubov V. Povkhova, Elena N. Pushkova, Roman O. Novakovskiy, Martin Pavelek, Gleb N. Vladimirov, Evgeny N. Nikolaev, Oxana A. Kovaleva, Yury I. Kostyukevich, Vitaliy V. Chagovets, Elena V. Romanova, Anastasiya V. Snezhkina, Anna V. Kudryavtseva, George S. Krasnov, Nataliya V. Melnikova

**Affiliations:** 1 Engelhardt Institute of Molecular Biology, Russian Academy of Sciences, Moscow, Russia; 2 Peoples’ Friendship University of Russia (RUDN University), Moscow, Russia; 3 Federal Research Center for Bast Fiber Crops, Torzhok, Russia; 4 All-Russian Horticultural Institute for Breeding, Agrotechnology and Nursery, Moscow, Russia; 5 Moscow Institute of Physics and Technology, Dolgoprudny, Russia; 6 Agritec, Plant Research LTD, Sumperk, Czech Republic; 7 Skolkovo Institute of Science and Technology, Moscow, Russia; 8 Kulakov National Medical Research Center for Obstetrics, Gynecology and Perinatology, Ministry of Healthcare of the Russian Federation, Moscow, Russia

**Keywords:** Flax, *Linum usitatissimum* L., *SAD*, *FAD*, Desaturases, Fatty acids, Genetic diversity, Polymorphism, Deep sequencing

## Abstract

**Background:**

Flax (*Linum usitatissimum* L.) is grown for fiber and seed in many countries. Flax cultivars differ in the oil composition and, depending on the ratio of fatty acids, are used in pharmaceutical, food, or paint industries. It is known that genes of *SAD* (stearoyl-ACP desaturase) and *FAD* (fatty acid desaturase) families play a key role in the synthesis of fatty acids, and some alleles of these genes are associated with a certain composition of flax oil. However, data on genetic polymorphism of these genes are still insufficient.

**Results:**

On the basis of the collection of the Institute for Flax (Torzhok, Russia), we formed a representative set of 84 cultivars and lines reflecting the diversity of fatty acid composition of flax oil. An approach for the determination of full-length sequences of *SAD1*, *SAD2*, *FAD2A*, *FAD2B*, *FAD3A*, and *FAD3B* genes using the Illumina platform was developed and deep sequencing of the 6 genes in 84 flax samples was performed on MiSeq. The obtained high coverage (about 400x on average) enabled accurate assessment of polymorphisms in *SAD1*, *SAD2*, *FAD2A*, *FAD2B*, *FAD3A*, and *FAD3B* genes and evaluation of cultivar/line heterogeneity. The highest level of genetic diversity was observed for *FAD3A* and *FAD3B* genes – 91 and 62 polymorphisms respectively. Correlation analysis revealed associations between particular variants in *SAD* and *FAD* genes and predominantly those fatty acids whose conversion they catalyze: *SAD* – stearic and oleic acids, *FAD2* – oleic and linoleic acids, *FAD3* – linoleic and linolenic acids. All except one low-linolenic flax cultivars/lines contained both the substitution of tryptophan to stop codon in the *FAD3A* gene and histidine to tyrosine substitution in the *FAD3B* gene, while samples with only one of these polymorphisms had medium content of linolenic acid and cultivars/lines without them were high-linolenic.

**Conclusions:**

Genetic polymorphism of *SAD* and *FAD* genes was evaluated in the collection of flax cultivars and lines with diverse oil composition, and associations between particular polymorphisms and the ratio of fatty acids were revealed. The achieved results are the basis for the development of marker-assisted selection and DNA-based certification of flax cultivars.

## Background

Flax (*Linum usitatissimum* L.) is grown for fiber and seed in many countries. In plants, flax seed is one of the leading sources of healthy ω-3 fatty acid (linolenic, LIN, C18:3), whose content in regular flax is about 50–60%. Seeds also contain linoleic (ω-6, LIO, C18:2), oleic (ω-9, OLE, C18:1), stearic (STE, C18:0), and palmitic (PAL, C16:0) acids [1, 2]. Linseed is used for the production of paints, varnishes, biodiesel, resins, and animal feeds, while flaxseed is used for functional food and pharmaceutical products. Flax cultivars differ in the fatty acid composition of oil: solin flax, unlike traditional, has a very low LIN content (less than 5%), rich in LIO, and is characterized by long-term storage that is important for food industry [1–4]. LIN and LIO are polyunsaturated fatty acids that cannot be synthesized in mammalian tissues [5]. For optimal health and disease prevention, in the diet, the ω-6/ω-3 ratio is required to be 1–3/1; and the content of oxidative stable monounsaturated OLE is also important for the production of stable and healthy oils and other products [3, 6–8].

Fatty acid desaturases, which make double bonds in a hydrocarbon chain, play a key role in the synthesis of fatty acids and determine the flax oil composition and, therefore, the direction of oil use [9]. Stearoyl-ACP desaturases (SAD) catalyze the conversion of STE to OLE; fatty acid desaturases 2 and 3 (FAD2 and FAD3) are responsible for desaturation of OLE into LIO and LIO into LIN respectively. FAD2 and FAD3 are bound with the membrane, while SAD are plastid-localized soluble desaturases [10, 11]. Two paralogous genes, *SAD1* and *SAD2*, have three exons and two introns with a total length of 2665 and 2592 bp respectively and encode 396-residue proteins; paralogous *FAD2A* and *FAD2B* are intronless with a length of 1137 bp (*FAD2A*) and 1149 bp (*FAD2B*) and encode proteins of 378 and 382 amino acid residues respectively; *FAD3A* and *FAD3B* are also paralogous with six exons and five introns with a total length of 3280 bp (*FAD3A*) and 3002 bp (*FAD3B*) and encode proteins of 392 and 391 amino acid residues respectively [10, 12]. *SAD1* and *SAD2* genes have differential expression in flax: mRNA level of *SAD1* was lower and more constant between seed development stages and genotypes than that of *SAD2* [12–14]. It was supposed that *FAD2B* plays role in LIO accumulation, while *FAD2A* is important for the complete conversion of OLE to LIO [12]. *FAD3A* and *FAD3B* expression correlated with the accumulation of linolenic acid and the mRNA level of *FAD3B* was higher than that of *FAD3A* [12, 15, 16]. However, in the work of Thambugala and Cloutier, for flax genotypes with identical isoforms of *SAD1, SAD2, FAD2A*, *FAD2B*, *FAD3A*, and *FAD3B* genes but diverse content of fatty acids (OLE – 11.4-21.8%, LIO – 9.9-14.9%, LIN – 53.7-71.9%), no correlations between the expression of desaturase genes and oil composition were revealed [14]. Polymorphisms in *SAD* and *FAD* genes were shown to be associated with the fatty acid composition [10, 17]. However, data on the genetic diversity of these genes and their influence on flax oil composition are still insufficient that complicates the development of the marker-assisted selection.

In the present work, we analyzed a representative set of flax cultivars and lines, reflecting the *L. usitatissimum* diversity of fatty acid composition, using deep targeted sequencing of *SAD1*, *SAD2*, *FAD2A*, *FAD2B*, *FAD3A*, and *FAD3B* genes. Such an approach enabled accurate identification of polymorphisms, evaluation of sample heterogeneity, and subsequent search for associations between DNA variants and flax oil composition.

## Results

### Polymorphisms in SAD and FAD genes

Sequences of *SAD1*, *SAD2*, *FAD2A*, *FAD2B*, *FAD3A*, and *FAD3B* genes were determined using deep sequencing in the collection of 84 *L. usitatissimum* cultivars and lines that have diverse content of PAL (from 5 to 7.6%), STE (from 2.7 to 6.4%), OLE (from 12.9 to 24%), LIO (from 11.9 to 72.4%), and LIN (from 2.7 to 65.3%) (Additional file 1). After library preparation, 40 amplicons about 600 bp in length, which contained target sequences, sequences that are necessary for processing on the Illumina platform, and dual-index barcodes, were obtained for each of 84 cultivars and lines and sequenced on MiSeq (Illumina, USA) in a 600-cycle format. On average, the coverage was 400x for each studied gene for each line and cultivar. The raw sequencing data were deposited in the Sequence Read Archive (SRA) under the accession number PRJNA625974.

Reads were trimmed and mapped to the *L. usitatissimum* genome (GCA_000224295.2/ASM22429v2) using BWA-MEM that demonstrated approximately 80% average overall read mapping rate, whereas bowtie2 showed only 50%. The search for polymorphisms was performed using VarScan and freeBayes. More than half of the found polymorphisms were characterized with very low variant allele frequency (VAF), 2–20%, throughout all the samples under analysis. This VAF is too low and these polymorphisms may represent either sequencing errors, PCR mispriming, amplification of off-target alleles not included in the genome assembly or other artifacts. Hence, we filtered out these variants based on VAF distribution analysis. VarScan enabled identification of 232 polymorphisms: *SAD1 *– 14, *SAD2 *– 14, *FAD2A* – 21, *FAD2B* – 11, *FAD3A* – 101, *FAD3B* – 71, while freeBayes revealed 239 polymorphisms: *SAD1 *– 15, *SAD2 *– 15, *FAD2A* – 31, *FAD2B* – 15, *FAD3A* – 90, *FAD3B* – 73. Most of the identified by VarScan and freeBayes variants were common: *SAD1 *– 12, *SAD2 *– 12, *FAD2A* – 20, *FAD2B* – 11, *FAD3A* – 91, *FAD3B* – 62 (according to the VarScan interpretation of polymorphisms). Thus, *FAD3A* and *FAD3B* genes were characterized by the highest genetic diversity. The detailed results are represented in Additional files 2, 3, 4, and 5 in the form of variant tables (allele frequencies for each identified polymorphism for each studied cultivar and line are reflected in columns named “alternative allele coverage”, “reference allele coverage”, and “alternative allele ratio”) and heatmaps (the content of PAL, STE, OLE, LIO, and LIN and the alternative allele ratio for each identified polymorphism are reflected in color scales for all studied cultivars and lines).

### Sample heterogeneity

**Fig. 1:**
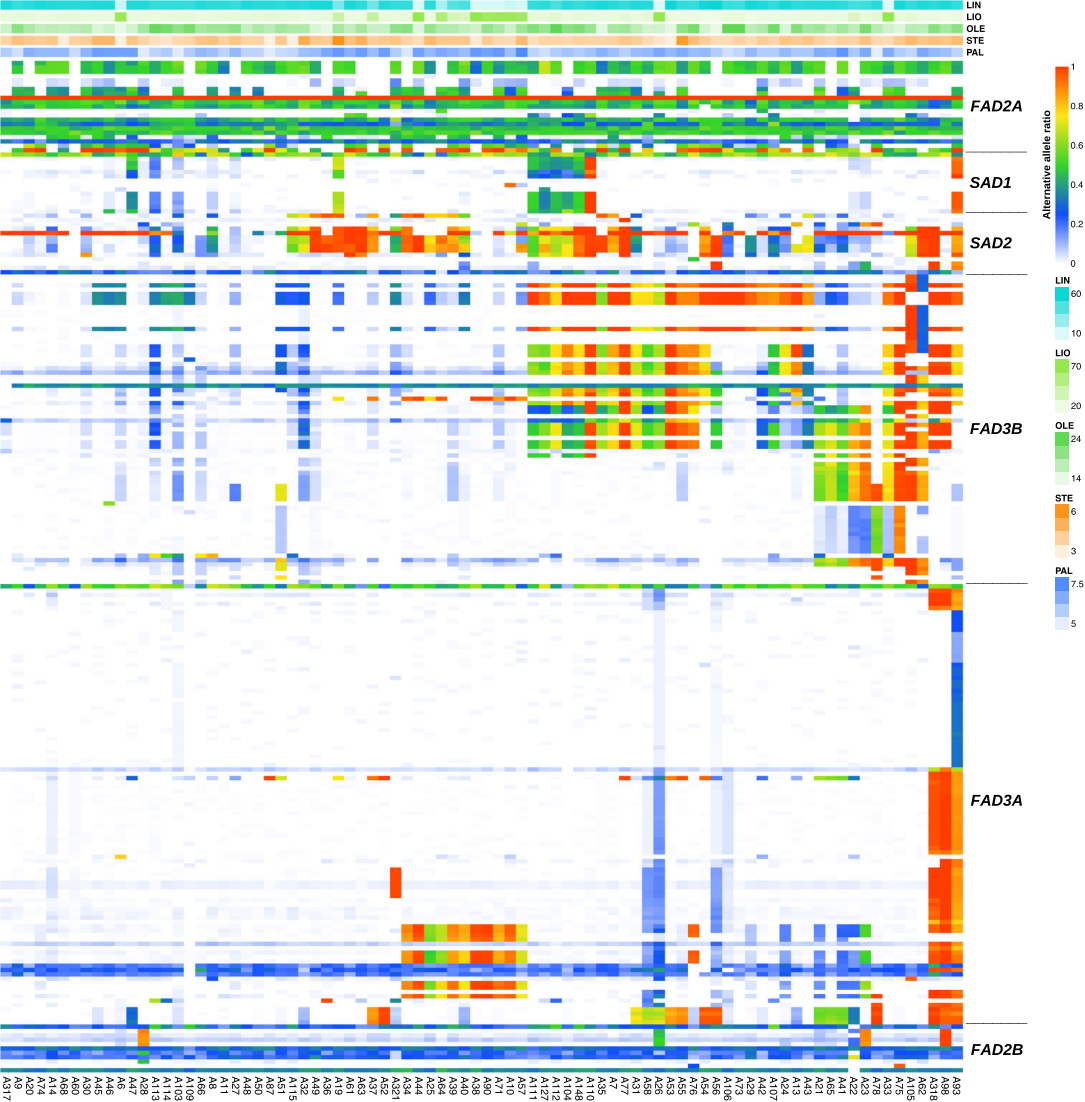
Heatmap reflecting polymorphisms in *SAD1*, *SAD2*, *FAD2A*, *FAD2B*, *FAD3A*, and *FAD3B* genes identified in 84 flax cultivars and lines. VarScan results are presented. Flax cultivars and lines are arranged along the horizontal axis and polymorphisms in *SAD1*, *SAD2*, *FAD2A*, *FAD2B*, *FAD3A*, and *FAD3B* genes are arranged along the vertical axis. The content of PAL (palmitic), STE (stearic), OLE (oleic), LIO (linoleic), and LIN (linolenic) and the alternative allele ratio for each identified polymorphism are reflected in color scales for all studied cultivars and lines

Our approach that is based on the sequencing of pools of plants (about 50 for each cultivar and line) enabled assessment of cultivar/line heterogeneity. We revealed that the majority of samples showed heterogeneity for at least one of the genes. Polymorphisms in the *FAD3B* gene is the most illustrative example of heterogeneity: for particular variants, in some samples, reference alleles prevailed, in other samples, alternative alleles prevailed, and samples with reference and alternative alleles with close ratios were also found (Fig. 1, Additional files 4 and 5).

### Correlation analysis

Correlation analysis between the content of PAL, STE, OLE, LIO, and LIN and VAF of identified polymorphisms in *SAD1*, *SAD2*, *FAD2A*, *FAD2B*, *FAD3A*, and *FAD3B* genes revealed associations between particular DNA variants and fatty acid composition of flax oil. In Table 1, data on polymorphisms with Spearman’s correlation coefficients more than 0.25 or less than −0.25 (*p-value* < 0.05) for both VarScan and freeBayes results are represented, and the results of correlation analysis for all identified by VarScan and freeBayes polymorphisms are represented in Additional files 6 and 7. In total, we found 31 variants associated with the content of one or several fatty acids (fatty acid associated polymorphisms, FAA polymorphisms): 29 SNPs (single nucleotide polymorphisms) and 2 indels (insertions/deletions). For the *SAD1* gene, 5 SNPs correlated with the oil composition, for *SAD2 – *2 SNPs and 1 indel, for *FAD2A* – 3 SNPs, for *FAD3A –* 9 SNPs, for *FAD3B* – 10 SNPs and 1 indel.

**Table 1:** Polymorphisms in *SAD1*, *SAD2*, *FAD2A*, *FAD3A*, and *FAD3B* genes associated with the fatty acid composition of flax oil

Site*	Location	Ref. allele	Alt. allele	Fatty acid**	VarScan		freeBayes	
					***r***_***s***_	***p-value***	***r***_***s***_	***p-value***
***SAD1*** (Chr. CP027620.1)								
2257330	Upstream	A	G	OLE	0.37	4 × 10^− 4^	0.37	5 × 10^− 4^
2257353	Upstream	T	C	OLE	0.35	0.001	0.33	0.002
2258938	Intron	C	G	OLE	0.27	0.01	0.34	0.002
2258980	Intron	T	C	OLE	0.34	0.002	0.37	6 × 10^−4^
2260202	Downstream	T	G	OLE	0.35	0.001	0.32	0.003
***SAD2*** (Chr. CP027621.1)								
17364707	Upstream	A	AG	OLE	0.31	0.004	0.30	0.005
17365164	Exon	T	C	STE	0.31	0.009	0.31	0.009
17367383	Downstream	G	T	LIO	−0.28	0.01	−0.28	0.01
***FAD2A*** (Chr. CP027619.1)								
5296364	Exon	G	A	PAL	−0.28	0.01	− 0.29	0.007
5296364	Exon	G	A	STE	−0.34	0.002	−0.34	0.002
5296658	Exon	C	T	STE	0.38	4 × 10^−4^	0.39	3 × 10^−4^
5296705	Downstream	C	A	PAL	0.35	0.006	0.35	0.005
***FAD3A*** (Chr. CP027631.1)								
16092241	Intron	T	C	LIN	−0.32	0.003	−0.35	0.001
16092241	Intron	T	C	PAL	0.29	0.008	0.28	0.009
16092273	Intron	C	A	LIN	−0.32	0.003	−0.37	4 × 10^−4^
16092294	Exon	T	C	LIN	−0.33	0.002	−0.39	2 × 10^− 4^
16092294	Exon	T	C	LIO	0.32	0.003	0.34	0.002
16092294	Exon	T	C	PAL	0.36	7 × 10^−4^	0.35	0.001
**16092348**	Exon	**C**	**T**	LIN	−0.29	0.008	−0.29	0.007
**16092348**	Exon	**C**	**T**	PAL	0.33	0.002	0.34	0.002
16092575	Exon	C	T	LIN	−0.36	8 × 10^−4^	−0.42	7 × 10^−6^
16092674	Upstream	G	T	LIN	−0.32	0.003	−0.38	3 × 10^−4^
16092741	Upstream	T	TAA	LIN	−0.37	5 × 10^−4^	− 0.36	8 × 10^− 4^
16093029	Upstream	T	C	LIN	−0.36	8 × 10^−4^	− 0.42	9 × 10^− 6^
16093029	Upstream	T	C	LIO	0.27	0.01	0.28	0.01
16093029	Upstream	T	C	PAL	0.39	3 × 10^−4^	0.40	2 × 10^− 4^
16093040	Upstream	C	A	LIN	−0.43	7 × 10^−6^	− 0.45	2 × 10^− 6^
16093040	Upstream	C	A	LIO	0.33	0.003	0.29	0.008
16093040	Upstream	C	A	PAL	0.35	0.001	0.33	0.002
***FAD3B*** (Chr. CP027622.1)								
1034358	Upstream	G	A	STE	0.28	0.01	0.26	0.02
1034389	Upstream	A	G	LIO	−0.31	0.005	−0.31	0.004
1034526	Upstream	C	A	LIO	−0.26	0.02	−0.25	0.02
1034873	Upstream	G	A	LIO	−0.32	0.004	−0.27	0.02
1034904	Upstream	G	C	LIO	−0.30	0.007	−0.30	0.006
1035028	Upstream	A	G	LIO	−0.28	0.01	−0.29	0.007
1035480	Exon	A	G	LIO	−0.29	0.007	−0.30	0.006
**1035655**	Exon	**C**	**T**	LIO	0.28	0.009	0.32	0.003
**1035655**	Exon	**C**	**T**	LIN	−0.37	6 × 10^−4^	−0.38	4 × 10^−4^
**1035655**	Exon	**C**	**T**	PAL	0.32	0.003	0.33	0.002
1035674	Exon	T	G	LIO	−0.26	0.02	−0.29	0.007
1036195	Intron	G	T	LIO	−0.26	0.02	−0.30	0.006
1037964	Intron	G	C	LIN	−0.26	0.02	−0.25	0.02

*Ref. allele* Reference allele, *Alt. allele* Alternative allele, *PAL* Palmitic acid, *STE* Stearic acid, *OLE* Oleic acid, *LIO* Linoleic acid, *LIN* Linolenic acid, *r*_*s*_ – Spearman’s rank correlation coefficient between the ratio of Alt. allele and the content of fatty acid. Polymorphisms with *r*_*s*_ < −0.25 or *r*_*s*_ > 0.25 for both VarScan and freeBayes results are presented. The key polymorphisms in *FAD3A* and *FAD3B* genes associated with LIN content in flax oil are shown in bold. * – the coordinates are given according to the *L.* *usitatissimum* genome assembly GCA_000224295.2/ASM22429v2. ** – fatty acid with the content of which the polymorphism is associated

The correlations were revealed between variants in desaturase genes and predominantly those fatty acids, in the biosynthesis of which they are involved: 5 polymorphisms in the *SAD1* gene were associated with OLE content, 2 in the *SAD2* gene – with the content of STE and OLE, 9 in *FAD3A* and 10 in *FAD3B* – with the content of LIO and LIN. However, 5 polymorphisms in *FAD3A* and 2 in *FAD3B* also correlated with PAL and STE content. For variants in the *FAD2A* gene, correlations were shown only with the content of PAL and STE, but no strong associations were observed with OLE and LIO content, in whose biosynthesis *FAD2A* is involved (see Table 1).

For the *SAD1* gene, for all five FAA polymorphisms, VAFs were either present in close to each other ratios or absent in each individual genotype, while for *SAD2,* we did not observe such patterns. For the *FAD2A* gene, VAFs of FAA polymorphisms ranged from 0.2 to 0.5 in most of the samples and diverged in individual samples. For the *FAD3A* gene, VAFs of all 9 associated with LIN content polymorphisms were close in most of the samples, however, in some samples, for sites 16092294, 16092348, 16093029, and 16093040, significant differences in VAFs were identified compared to the other sites: for samples A58, A26, A56, A318, A98, and A93 – for all four sites, for A76, A22, and A23 – only for site 16092348 (see Additional file 8). It should be noted that these sites were also associated with LIO content. The greatest diversity in VAFs of FAA polymorphisms in individual samples was revealed for *FAD3B*.

### Amino acid substitutions

In the *SAD1* gene, FAA SNPs were identified in an intron (2), before the gene, i.e. upstream (2), and after the gene, i.e. downstream (1). In the *SAD2* gene, FAA polymorphisms were located in an exon (replacement of GGT with AGT in CP027621.1:17365164 results in substitution of glycine to serine), an intron, and after the gene. In the *FAD2A* gene, two SNPs were found in an exon, but none of them results in the substitution of amino acids, and one – after the gene. In the *FAD3A* gene, two FAA SNPs result in substitutions of amino acids: alanine to threonine (CP027631.1:16092575) and tryptophan to stop codon (CP027631.1:16092348). In the *FAD3B* gene, replacement of CAT with TAT results in substitution of histidine to tyrosine (CP027622.1:1035655) and replacement of ATC with AGC results in substitution of serine to isoleucine (CP027622.1:1035674). Other FAA polymorphisms in *FAD3A* and *FAD3B* genes do not lead to changes in the amino acid sequences.

### Clustering of studied cultivars and lines

**Fig. 2:**
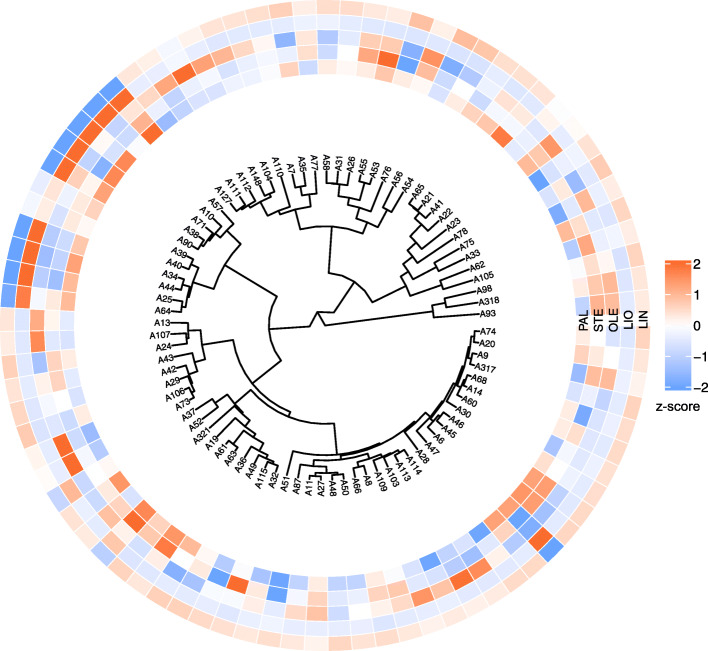
Clusterization of 84 flax cultivars and lines based on the polymorphisms in *SAD1*, *SAD2*, *FAD2A*, *FAD2B*, *FAD3A*, and *FAD3B* genes. VarScan results were used for clusterization. Color scales reflect the content of fatty acids: PAL – palmitic, STE – stearic, OLE – oleic, LIO – linoleic, LIN – linolenic

Clustering of the studied flax cultivars and lines was performed based on the polymorphisms in *SAD1*, *SAD2*, *FAD2A*, *FAD2B*, *FAD3A*, and *FAD3B* genes, which were identified using VarScan and freeBayes (Fig. 2 and Additional file 9). The results of clustering based on VarScan and freeBayes data were very similar. The cluster that included 9 out of 10 cultivars and lines with low LIN and high LIO content (A57, A10, A71, A38, A90, A34, A44, A25, and A64, see Fig. 2) was revealed. However, one low-LIN sample (A6) was not located in this cluster. For other clusters, no clear associations with the fatty acid composition were identified that could be due to the predominance of polymorphisms in *FAD3A* and *FAD3B* genes, which are involved in the desaturation of LIO into LIN.

The analysis of variants in *FAD3A* and *FAD3B* was performed for the A6 low-LIN sample, which was out of the cluster of other low-LIN cultivars and lines. We found the SNP in the *FAD3A* gene (site CP027631.1:16090340) that results in the replacement of arginine with a stop codon that can explain the low content of LIN. Such SNP was also identified in A22, A42, and A64 samples, however, with low VAF, about 5–10%, while only in A6, about 80% of plants had this SNP (Additional files 2 and 3).

**Fig. 3:**
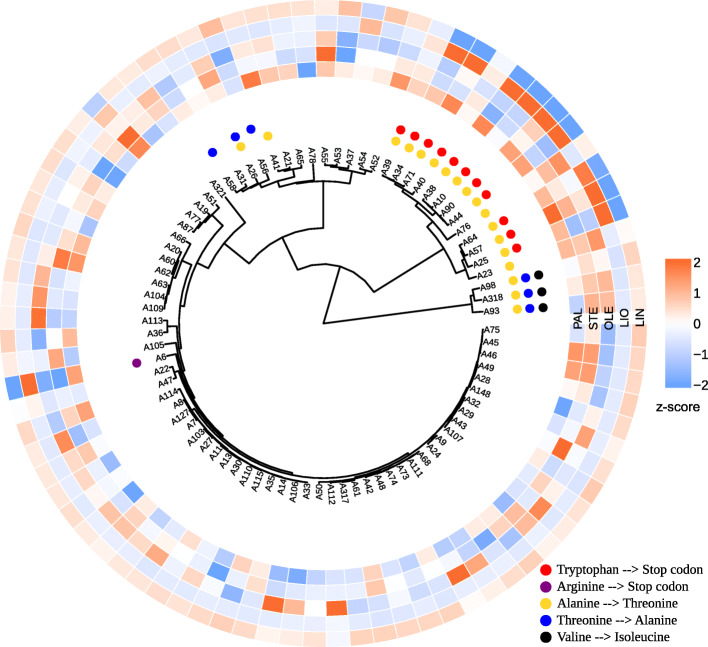
Clusterization of 84 flax cultivars and lines based on the polymorphisms in the *FAD3A* gene. VarScan results were used for clusterization. Color scales reflect the content of fatty acids: PAL – palmitic, STE – stearic, OLE – oleic, LIO – linoleic, LIN – linolenic. Missense and nonsense mutations are indicated with colored dots

**Fig. 4:**
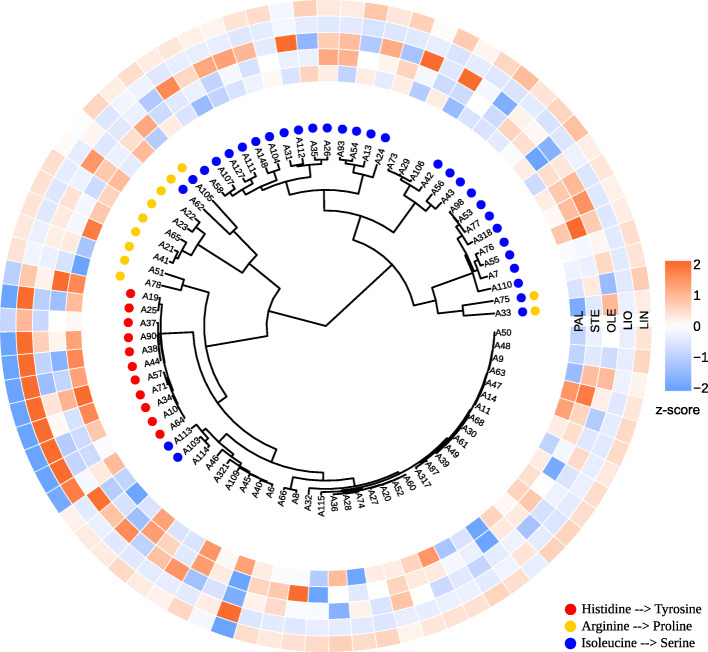
Clusterization of 84 flax cultivars and lines based on the polymorphisms in the *FAD3B* gene. VarScan results were used for clusterization. Color scales reflect the content of fatty acids: PAL – palmitic, STE – stearic, OLE – oleic, LIO – linoleic, LIN – linolenic. Missense and nonsense mutations are indicated with colored dots

We also performed the clustering of the flax samples based on the polymorphisms in individual genes: *SAD1*, *SAD2*, *FAD2A*, *FAD2B*, *FAD3A*, and *FAD3B*. Results are represented in Additional file 10 (VarScan) and Additional file 11 (freeBayes). Polymorphisms identified by both variant callers gave similar dendrograms for the studied genes. The clustering revealed that *FAD3A* and *FAD3B* polymorphisms were responsible for the cluster that included most of the low-LIN flax cultivars and lines (Figs. 3 and 4).

As can be seen from Figs. 3 and 4, all low-LIN samples from the low-LIN cluster contained both the stop codon (CP027631.1:16092348) in the *FAD3A* gene and histidine to tyrosine substitution (CP027622.1:1035655) in the *FAD3B* gene. Samples with only one of these polymorphisms were mid-LIN (32.6–46.8%): A39 and A40 samples contained only the stop codon in *FAD3A* (Fig. 3), while A19 and A37 samples – only histidine to tyrosine substitution in *FAD3B* (Fig. 4). All the samples without these polymorphisms were high-LIN except low-LIN A6, which has the arginine to stop codon substitution in *FAD3A*.

Further analysis of FAA polymorphisms in the *FAD3A* gene revealed that all low-LIN and mid-LIN samples with the SNP in site CP027631.1:16092348, which results in the tryptophan to stop codon substitution, also contained the rest 8 *FAD3A* FAA SNPs (Additional file 8). However, there were two samples, A76 and A23, that carried only these 8 FAA polymorphisms and were characterized by high LIN content. This points to the crucial role of tryptophan to stop codon substitution in the formation of low LIN content in flax cultivars and lines. The majority of *FAD3B* FAA polymorphisms showed a negative correlation with the content of LIO that indicates that they were presented in high-LIN samples, but as can be seen from Additional file 8, more than a third of high-LIN samples did not contain any of these variants. The only FAA polymorphism in the *FAD3B* gene with the negative correlation with LIN content is SNP in site CP027622.1:1035655 leading to the histidine to tyrosine substitution. Therefore, this SNP, which is presented in low-LIN and mid-LIN samples in the absence of the rest 10 *FAD3B* FAA variants, plays a key role in providing the low content of linolenic acid in flax oil.

## Discussion

In the present study, deep sequencing was used to assess the genetic diversity of *SAD1*, *SAD2*, *FAD2A*, *FAD2B*, *FAD3A*, and *FAD3B* genes, which are known to be involved in fatty acid biosynthesis, in the collection of 84 flax cultivars and lines with different fatty acid composition. DNA was extracted from about 50 seedlings for each variety and the high coverage enabled accurate identification of DNA polymorphisms even in the case of cultivar/line heterogeneity. Draft genome sequence of flax was obtained in 2012 [18], and in 2018, chromosome-scale pseudomolecules were received using BioNano genome optical mapping, and the genome assembly was improved [19]. These results allowed us to map the Illumina reads obtained for *SAD* and *FAD* genes to the *L. usitatissimum* genome (GCA_000224295.2/ASM22429v2) thus reducing the probability of misidentification of polymorphisms in the studied genes due to mispriming and sequencing of others fatty acid-related genes or pseudogenes with high homology at the DNA level [20].

Two widely used variant callers, VarScan and freeBayes, were applied for polymorphism identification in *SAD1*, *SAD2*, *FAD2A*, *FAD2B*, *FAD3A*, and *FAD3B* genes of studied flax cultivars and lines. The highest genetic diversity was revealed for *FAD3A* and *FAD3B* genes – 91 and 62 polymorphisms respectively. This result is consistent with the data of other researches [10, 21]. *FAD3A* and *FAD3B* desaturate LIO into LIN [15, 16] and flax is characterized by a wide genetic diversity for LIO and LIN content [10]. In the studied collection of *L. usitatissimum*, the variability of LIN and LIO content was the highest (24- and 6-fold difference respectively) compared to OLE, STE, and PAL content (from 1.5- to 2.4-fold difference) and this can be the reason of identification of numerous polymorphisms in *FAD3A* and *FAD3B* genes.

Correlation analysis allowed the determination of variants that were associated with the composition of flax oil (Table 1, Additional files 6, 7 and 8). Most correlations were identified between polymorphisms in *SAD* and *FAD* genes and the content of those fatty acids in whose biosynthesis they are involved: *SAD* variants were predominantly associated with STE and OLE content, *FAD2* – OLE and LIO, *FAD3* – LIO and LIN. However, some associations with other fatty acids were also revealed. Similar results were obtained in the study of Thambugala et al., in which variants in *SAD2* were significant for OLE content; *FAD2A* – for OLE and LIO; *FAD3A* – for LIN, LIO, and PAL; *FAD3B* – for LIN, LIO, OLE, and PAL [10]. In a genome-wide association study of flax, correlations were observed between *FAD3A*, *FAD3B* and LIN, LIO and between *SAD1* and STE [17]. In the present study, Spearman’s correlation coefficient varied from −0.43 to 0.39, and for further analysis, more than 0.25 or less than −0.25 coefficients were considered significant. Most of such polymorphisms were identified in *FAD3A* and *FAD3B* genes. It should be noted that for all associated with oil composition polymorphisms, at least one and more often several studied flax samples were heterogeneous. This indicates that in one flax cultivar or line, genotypes with different fatty acid composition could be present and the use of molecular markers for *SAD* and *FAD* genes can be useful to increase the cultivar purity.

The identified FAA polymorphisms were located in different regions of the genes – promoters, exons, and introns. The search for amino acid substitutions caused by these polymorphisms in *SAD* and *FAD* genes revealed missense mutations in *SAD2*, *FAD3A*, and *FAD3B* genes and the nonsense mutation in *FAD3A*. However, most of the discovered polymorphisms were silent. Our data are similar to the results of previous studies, where silent and missense mutations were identified in *SAD*, *FAD2,* and *FAD3* genes, while stop codons were revealed in *FAD3* [10, 12, 15, 16, 22]. In the present study, most of the samples with low LIN and high LIO content had both the mutation in CP027631.1:16092348 that resulted in a stop codon in *FAD3A* and the mutation in CP027622.1:1035655 that resulted in histidine to tyrosine substitution in *FAD3B*, while all cultivars and lines with high LIN had not these mutations. A39 and A40 samples with 43.5 and 46.8% LIN had only the stop codon in *FAD3A*, while A19 and A37 with 32.6 and 35.0% LIN had only the histidine to tyrosine substitution in *FAD3B*. So, all four samples characterized with only one of the two mutations were mid-LIN. The A6 sample is the exclusion from the general trend: this cultivar is low-linolenic (9.4% LIN) and has none of the above mutations in *FAD3A* and *FAD3B* genes. In A6, another mutation in *FAD3A* resulting in a stop codon was identified in site CP027631.1:16090340, and for this cultivar, this mutation is the likely reason for low LIN content, however, no polymorphisms with at least 40% VAF were found in *FAD3B*.

Although we revealed a large number of polymorphisms in *FAD3A* and *FAD3B* genes and found among them a significant number of those associated with the fatty acid composition (9 for *FAD3A* and 11 for *FAD3B*), the further analysis of the obtained data showed that only three variants play a key role in the determination of the ratio of linoleic and linolenic acids in our sample set – SNPs in sites CP027631.1:16092348, CP027631.1:16090340, and CP027622.1:1035655 resulting in stop codons in the *FAD3A* gene and histidine to tyrosine substitution in *FAD3B*. The presence or absence of these polymorphisms can result in high, medium, or low content of linolenic acid that determines the use of flax oil. The rest eight FAA SNPs in the *FAD3A* gene were presented in a sample all together either with the key one (CP027631.1:16092348) or without it that points to the common origin of these cultivars and lines. On the contrary, FAA polymorphisms in *FAD3B* were presented in a sample by either the key one (CP027622.1:1035655) or different combinations of the rest ten. These findings are useful for the breeding of cultivars with a given LIN content: if one of the parents in a breeding program is low-LIN while another one is high-LIN, one can select families that are homozygous for *FAD3A* and *FAD3B* genes and have allele variants that correspond to high, low, or medium LIN content already among F_2_ or F_3_ progeny using molecular markers that will increase the efficiency of the breeding process.

## Conclusions

We evaluated the genetic polymorphism of *SAD1*, *SAD2*, *FAD2A*, *FAD2B*, *FAD3A*, and *FAD3B* genes, which play a key role in the desaturation of fatty acids in flax seed. Deep Illumina sequencing allowed us to obtain high coverage and accurately identify SNPs and indels in the studied genes taking into account cultivar and line heterogeneity. The highest genetic diversity was observed for *FAD3A* and *FAD3B* genes. Correlation analysis revealed DNA variants in *SAD1*, *SAD2*, *FAD2A*, *FAD3A*, and *FAD3B* that were associated with the content of PAL, STE, OLE, LIO, and LIN. The majority of low-LIN samples were characterized by both tryptophan to stop codon substitution in *FAD3A* and replacement of histidine with tyrosine in *FAD3B*, while the presence of only one of these polymorphisms was associated with medium LIN content. The rest low-LIN sample contained arginine to stop codon substitution in *FAD3A* that was inherent only to this cultivar from the analyzed collection. The identified polymorphisms could be used for the development of molecular markers for the selection of valuable genotypes in flax breeding and obtaining pure improved cultivars that can significantly increase the efficiency of breeding programs and also help in DNA-based certification of flax cultivars. Besides, data on polymorphisms in *SAD* and *FAD* genes and their associations with the oil composition could be useful for the development of CRISPR-based approaches for the directional changes in the fatty acid composition of flax oil.

## Methods

### Plant material

A total of 84 flax cultivars and lines from the collection of Institute for Flax (Torzhok, Russia) with diverse content of PAL, STE, OLE, LIO, and LIN were used in the present work (Additional file 1). The oil content and composition were assessed as described in Bjelková et al. [23] according to the Czech Office for Standards, Metrology, and Testing.

Seeds were sterilized in 70% ethanol for 1 min and in 1% sodium hypochlorite for 10 min and then grown on a filter paper in Petri dishes for 5 days. For each cultivar and line, about 50 seedlings were yielded and used for DNA extraction with a CTAB method with minor modifications [24]. A DNA concentration was assessed using the Qubit 2.0 fluorometer (Life Technologies, USA).

### Preparation and sequencing of amplicon libraries

For sequencing of *SAD1*, *SAD2, FAD2A*, *FAD2B, FAD3A,* and *FAD3B* genes, we used modified Illumina protocol for library preparation (https://support.illumina.com/documents/documentation/chemistry_documentation/16s/16s-metagenomic-library-prep-guide-15044223-b.pdf). Studied genes with the promoters were divided into regions about 450–500 bp in length in such a way that primers were designed for less variable sites and, if possible, one pair of primers can amplify regions of *SAD1* and *SAD2, FAD2A* and *FAD2B*, or *FAD3A* and *FAD3B* simultaneously. MEGA software [25] was used for the analysis of *SAD* and *FAD* sequences from GenBank and our data on flax genomes and transcriptomes (unpublished). NCBI Primer-BLAST [26] and MethyMer [27] were applied for primer design. As a result, 12 primer pairs for *SAD* genes, 10 for *FAD2* genes, and 18 for *FAD3* genes were developed (Additional file 12). Two-stage PCR was performed for library preparation as described by us earlier [28]. In brief, the first stage allowed amplification of target sequences and appending of overhang adapters, while in the second stage, Nextera XT v2 index primers containing sequencing adapters and dual-index barcodes were used. To increase the specificity, touchdown PCR was used in the first stage. For each studied gene, before the second stage of PCR, the amplicons were combined for each flax cultivar and line by taking 4 μl per sample. The quality and concentration of the prepared amplicon libraries were evaluated on Agilent 2100 bioanalyzer (Agilent, USA) and Qubit 2.0 (Life Technologies). Then, the libraries were normalized, pooled, and sequenced on MiSeq (Illumina, USA) in a 600-cycle format (paired 300-bp reads, v3 reagents).

### Deep sequencing data processing and variant calling

Illumina reads were trimmed and filtered with trimmomatic [29]. Then reads were mapped to the *L. usitatissimum* reference genome (assembly GCA_000224295.2/ASM22429v2). We decided to map reads to the full-length genome assembly instead of a set of PCR target regions. The main reason is possible PCR mispriming and amplification of off-target homologous genes, which can produce reads that significantly differ from the target genes, and these differences may be considered as SNPs/indels by a variant caller.

We used BWA-MEM [30] as it allows more mismatches and indels compared to bowtie2. This is especially important for us since we deal with flax cultivars and lines, whose genomes may significantly differ from the reference assembly.

The mapped reads were reordered and grouped with Picard tools. We did not mark duplicates as it is not applicable for PCR amplicon sequencing. Finally, we performed variant calling by two different tools: VarScan [31] and freeBayes [32]. Variants with VAF low than 20% in all the samples were filtered out.

Next, we analyzed associations between the content of PAL, STE, OLE, LIO, LIN and polymorphisms in *SAD1, SAD2, FAD2A, FAD2B, FAD3A, FAD3B* genes by calculating Spearman’s and Kendall’s rank correlation coefficients and *p-values*. We paid special attention to the value of relative standard deviation (rStD) of VAF values. The greater the rStD, the more reliable the association.

The search for amino acid substitutions caused by FAA polymorphisms in *SAD* and *FAD* genes was performed using our flax genome and transcriptome sequencing data (unpublished) to estimate the exon-intron boundaries.

Finally, we clustered the samples based on their genotype (i.e. VAF profile; per each gene or across all the genes). Before that, we zeroed all VAF < 20% to reduce the impact of remaining off-targets and calculated inter-sample Euclidean distance. Clustering was performed by the Ward D2 method. All calculations were performed in the R environment. Visualization of clustering dendrograms and heatmaps was performed using pheatmap, d3heatmap, ggtree, and gheatmap packages.

## Supplementary information

**Additional file 1.** Characteristics of 84 flax cultivars and lines.

**Additional file 2. **Polymorphisms of *SAD1*, *SAD2*, *FAD2A*, *FAD2B*, *FAD3A*, and *FAD3B* genes identified in 84 flax cultivars and lines using VarScan.

**Additional file 3. **Polymorphisms of *SAD1*, *SAD2*, *FAD2A*, *FAD2B*, *FAD3A*, and *FAD3B* genes identified in 84 flax cultivars and lines using freeBayes.

**Additional file 4. **Heatmap reflecting polymorphisms of *SAD1*, *SAD2*, *FAD2A*, *FAD2B*, *FAD3A*, and *FAD3B* genes identified in 84 flax cultivars and lines using VarScan. Flax cultivars and lines are arranged along the horizontal axis and polymorphisms in *SAD1*, *SAD2*, *FAD2A*, *FAD2B*, *FAD3A*, and *FAD3B* genes are arranged along the vertical axis. The content of PAL (palmitic), STE (stearic), OLE (oleic), LIO (linoleic), and LIN (linolenic) and the alternative allele ratio (Alt. ratio) for each identified polymorphism are reflected in color scales for all studied cultivars and lines.

**Additional file 5. **Heatmap reflecting polymorphisms of *SAD1*, *SAD2*, *FAD2A*, *FAD2B*, *FAD3A*, and *FAD3B* genes identified in 84 flax cultivars and lines using freeBayes. Flax cultivars and lines are arranged along the horizontal axis and polymorphisms in *SAD1*, *SAD2*, *FAD2A*, *FAD2B*, *FAD3A*, and *FAD3B* genes are arranged along the vertical axis. The content of PAL (palmitic), STE (stearic), OLE (oleic), LIO (linoleic), and LIN (linolenic) and the alternative allele ratio (Alt. ratio) for each identified polymorphism are reflected in color scales for all studied cultivars and lines.

**Additional file 6. **Correlations between polymorphisms in *SAD1*, *SAD2*, *FAD2A*, *FAD2B*, *FAD3A*, and *FAD3B* genes revealed by VarScan and fatty acid composition of flax oil.

**Additional file 7. **Correlations between polymorphisms in *SAD1*, *SAD2*, *FAD2A*, *FAD2B*, *FAD3A*, and *FAD3B* genes revealed by freeBayes and fatty acid composition of flax oil.

**Additional file 8. **Fatty acid associated polymorphisms in *SAD1*, *SAD2*, *FAD2A*, *FAD2B*, *FAD3A*, and *FAD3B* genes revealed by both VarScan and freeBayes in 84 flax cultivars and lines.

**Additional file 9. **Clusterization of 84 flax cultivars and lines based on polymorphisms in *SAD1*, *SAD2*, *FAD2A*, *FAD2B*, *FAD3A*, and *FAD3B* genes revealed by freeBayes.

**Additional file 10. **Clusterization of 84 flax samples based on polymorphisms in individual genes (*SAD1*, *SAD2*, *FAD2A*, *FAD2B*, *FAD3A*, and *FAD3B*) revealed by VarScan.

**Additional file 11. **Clusterization of 84 flax samples based on polymorphisms in individual genes (*SAD1*, *SAD2*, *FAD2A*, *FAD2B*, *FAD3A*, and *FAD3B*) revealed by freeBayes.

**Additional file 12.** Primers for the first stage of DNA library preparation.

## Data Availability

The dataset generated and analyzed during the current study is available in the Sequence Read Archive (SRA), PRJNA625974.

## Abbreviations

SAD: Stearoyl-ACP desaturase
FAD: Fatty acid desaturase
PAL: Palmitic acid
STE: Stearic acid
OLE: Oleic acid
LIO: Linoleic acid
LIN: Linolenic acid
VAF: Variant allele frequency
SNP: Single nucleotide polymorphism
FAA: Fatty acid associated
