# Supplementary material for: Genetic diversity of SAD and FAD genes responsible for the fatty acid composition in flax cultivars and lines
Source: BMC Plant Biol. 2020 Oct 14;20(Suppl 1):301. doi: 10.1186/s12870-020-02499-w (PMC7557025; doi:10.1186/s12870-020-02499-w)
Supplement: Supplementary file 1 — Additional file 1. Characteristics of 84 flax cultivars and lines. [file 12870_2020_2499_MOESM1_ESM.docx]

**Additional file 1. Characteristics of 84 flax cultivars and lines.**

| **Sample**  **number** | **Sample name** | **Palmitic (PAL), %** | **Stearic (STE), %** | **Oleic, (OLE), %** | **Linoleic, (LIO), %** | **Linolenic, (LIN), %** |
| --- | --- | --- | --- | --- | --- | --- |
| A6 | Lola | 7.0 | 2.7 | 12.9 | 68.0 | 9.4 |
| A7 | Recital | 6.2 | 3.9 | 18.1 | 14.7 | 57.1 |
| A8 | Oural | 6.2 | 4.5 | 19.2 | 15.0 | 55.1 |
| A9 | Baikal | 5.4 | 4.6 | 20.4 | 17.1 | 52.5 |
| A10 | Jantar | 6.2 | 3.5 | 15.3 | 66.8 | 8.3 |
| A11 | Bethune | 5.7 | 3.9 | 19.2 | 16.2 | 55.0 |
| A13 | Bachmalskij 1056 | 6.0 | 4.1 | 21.0 | 13.6 | 55.5 |
| A14 | Atalante | 6.4 | 4.3 | 16.4 | 14.7 | 58.2 |
| A19 | AGT 422/10 | 7.2 | 6.4 | 18.3 | 35.5 | 32.6 |
| A20 | AGT 439/10 | 6.6 | 4.2 | 17.8 | 15.9 | 55.4 |
| A21 | AGT 461/10 | 6.7 | 3.9 | 18.9 | 13.6 | 56.8 |
| A22 | AGT 464/10 | 6.0 | 3.8 | 16.4 | 24.9 | 48.9 |
| A23 | AGT 494/10 | 6.8 | 4.5 | 21.8 | 16.0 | 50.9 |
| A24 | AGT 545/10 | 6.4 | 4.4 | 14.2 | 14.3 | 60.6 |
| A25 | AGT 316/02 | 6.8 | З.1 | 14.9 | 72.4 | 2.7 |
| A26 | Benvanuto Real | 6.4 | 4.8 | 19.7 | 14.2 | 55.0 |
| A27 | Entre-Rios | 6.4 | 4.4 | 20.1 | 16.5 | 52.5 |
| A28 | Vitagold | 5.1 | 3.7 | 18.3 | 14.8 | 58.1 |
| A29 | Astral | 6.4 | 3.5 | 16.4 | 12.5 | 61.9 |
| A30 | Kaolin | 6.5 | 4.0 | 17.1 | 15.0 | 57.4 |
| A31 | Alaska | 6.3 | 4.0 | 16.6 | 15.3 | 57.8 |
| A32 | Baladin | 6.2 | 5.4 | 17.5 | 14.0 | 56.9 |
| A33 | Omega | 5.2 | 3.7 | 20.5 | 16.8 | 53.8 |
| A34 | Amon | 6.7 | 3.5 | 14.8 | 71.8 | 3.1 |
| A35 | Bilton | 6.6 | 3.8 | 13.4 | 15.5 | 60.7 |
| A36 | Prairie Blue | 6.8 | 4.0 | 13.1 | 15.6 | 60.5 |
| A37 | Raciol | 6.3 | 4.0 | 15.5 | 39.2 | 35.0 |
| A38 | AGT 1538/07 | 7.2 | 2.9 | 15.2 | 71.5 | 3.2 |
| A39 | AGT 1568/07 | 6.6 | 4.2 | 17.1 | 28.6 | 43.5 |
| A40 | AGT 467/08 | 6.3 | 4.2 | 16.1 | 26.7 | 46.8 |
| A41 | AGT 470/08 | 7.3 | 3.9 | 18.6 | 14.3 | 55.9 |
| A42 | AGT 510/08 | 5.9 | 4.0 | 17.4 | 13.8 | 59.0 |
| A43 | AGT 302/10 | 6.0 | 4.4 | 18.3 | 15.4 | 56.0 |
| A44 | AGT 368/10 | 7.0 | 3.4 | 13.9 | 72.3 | 3.5 |
| A45 | AGT 390/10 | 7.1 | 4.9 | 14.1 | 14.9 | 59.0 |
| A46 | AGT 399/10 | 7.0 | 4.9 | 15.1 | 14.4 | 58.6 |
| A47 | AGT 409/10 | 7.0 | 4.4 | 14.7 | 15.4 | 58.6 |
| A48 | AGT 306/10 | 5.7 | 3.7 | 15.7 | 15.8 | 59.2 |
| A49 | AGT 305/10 | 6.3 | 4.2 | 14.2 | 15.0 | 60.3 |
| A50 | AGT 307/10 | 5.7 | 3.8 | 17.8 | 16.6 | 56.1 |
| A51 | AGT 308/10 | 5.5 | 4.2 | 18.4 | 16.8 | 55.1 |
| A52 | AGT 393/10 | 7.1 | 4.2 | 15.6 | 16.8 | 56.3 |
| A53 | AGT 427/10 | 6.4 | 2.7 | 13.1 | 13.4 | 64.3 |
| A54 | IDG 4102 | 6.4 | 4.0 | 14.6 | 12.1 | 62.9 |
| A55 | IDG 4101 | 6.7 | 6.0 | 19.8 | 13.6 | 53.8 |
| A56 | IDG 4105 | 5.9 | 3.5 | 12.9 | 12.5 | 65.3 |
| A57 | AGT 1535/07 | 7.6 | 4.7 | 18.8 | 64.0 | 4.9 |
| A58 | Leane | 6.4 | 3.5 | 14.6 | 17.8 | 57.7 |
| A60 | McDuff | 5.5 | 3.9 | 16.5 | 17.3 | 56.8 |
| A61 | LF-L37 | 6.6 | 4.5 | 16.0 | 13.0 | 59.9 |
| A62 | Jeager | 6.8 | 4.3 | 16.5 | 13.2 | 59.2 |
| A63 | Natural | 7.1 | 5.2 | 18.4 | 14.0 | 55.2 |
| A64 | AGT 987/02 | 6.8 | 4.2 | 17.4 | 53.1 | 18.5 |
| A65 | AGT 583/05 | 6.6 | 3.5 | 17.0 | 15.7 | 57.2 |
| A66 | LS-L68 | 6.4 | 4.4 | 18.3 | 13.4 | 57.5 |
| A68 | Kinelskij 2000 | 6.4 | 2.8 | 16.2 | 17.0 | 57.6 |
| A71 | l. 1-1 Walaga | 7.2 | 3.6 | 18.0 | 66.8 | 4.5 |
| A73 | l. 1-3-2 Makovi M.A.G. | 5.6 | 3.9 | 24.0 | 16.1 | 50.5 |
| A74 | l. 1-2 Bolley Golden | 6.4 | 4.0 | 16.2 | 13.5 | 59.9 |
| A75 | l. 1-1 Mermilloid | 5.9 | 4.2 | 13.8 | 14.5 | 61.5 |
| A76 | l. 1-1-2 Rabat 12 | 6.3 | 4.6 | 22.0 | 13.9 | 53.2 |
| A77 | l. 1-1 Ottawa 2152 | 5.8 | 4.3 | 18.7 | 17.6 | 53.6 |
| A78 | l. 1-1-1 Liral Dominion | 5.0 | 3.6 | 16.9 | 18.2 | 56.3 |
| A87 | l. 1-1-1 Bionda | 5.1 | 2.7 | 16.7 | 18.3 | 57.2 |
| A90 | l. 1-1-2 Eyre | 7.0 | 4.1 | 19.6 | 64.8 | 4.5 |
| A93 | Cian | 5.7 | 4.7 | 20.3 | 14.9 | 54.3 |
| A98 | l. 1 k-1352 | 6.2 | 4.3 | 18.1 | 11.9 | 59.5 |
| A103 | Culbert | 5.1 | 3.6 | 20.1 | 16.8 | 54.3 |
| A104 | # 3857 | 6.1 | 3.6 | 20.6 | 13.7 | 56.0 |
| A105 | Eurodor | 5.6 | 4.8 | 16.9 | 18.8 | 53.9 |
| A106 | LM 96 | 5.4 | 3.5 | 23.7 | 15.3 | 52.1 |
| A107 | LM 92 | 5.9 | 3.7 | 22.3 | 14.1 | 54.1 |
| A109 | Clark | 5.9 | 3.7 | 21.9 | 16.0 | 52.6 |
| A110 | # 3814 | 6.1 | 3.8 | 19.3 | 14.7 | 56.0 |
| A111 | # 3872 | 5.7 | 3.4 | 21.2 | 16.5 | 53.2 |
| A112 | LM 91 | 5.9 | 3.8 | 23.8 | 19.4 | 47.0 |
| A113 | KL 24 | 5.7 | 3.3 | 23.2 | 14.9 | 52.9 |
| A114 | Norlin | 5.9 | 3.1 | 22.2 | 14.5 | 54.3 |
| A115 | M 2965 | 5.6 | 2.7 | 17.5 | 16.0 | 58.3 |
| A127 | Ilim | 5.4 | 3.8 | 19.3 | 16.2 | 55.2 |
| A148 | # 3871 | 5.9 | 3.9 | 21.4 | 16.3 | 52.4 |
| A317 | McBeth | 5.9 | 3.8 | 15.3 | 15.2 | 59.8 |
| A318 | Eole | 6.4 | 4.5 | 20.5 | 13.2 | 55.4 |
| A321 | Flanders | 5.9 | 4.4 | 20.2 | 15.1 | 54.4 |

*Note*: Cultivars and lines were provided by the Institute for Flax (Torzhok, Russia).
