# Supplementary figures and images for: Genetic diversity of SAD and FAD genes responsible for the fatty acid composition in flax cultivars and lines
Source: BMC Plant Biol. 2020 Oct 14;20(Suppl 1):301. doi: 10.1186/s12870-020-02499-w (PMC7557025; doi:10.1186/s12870-020-02499-w)

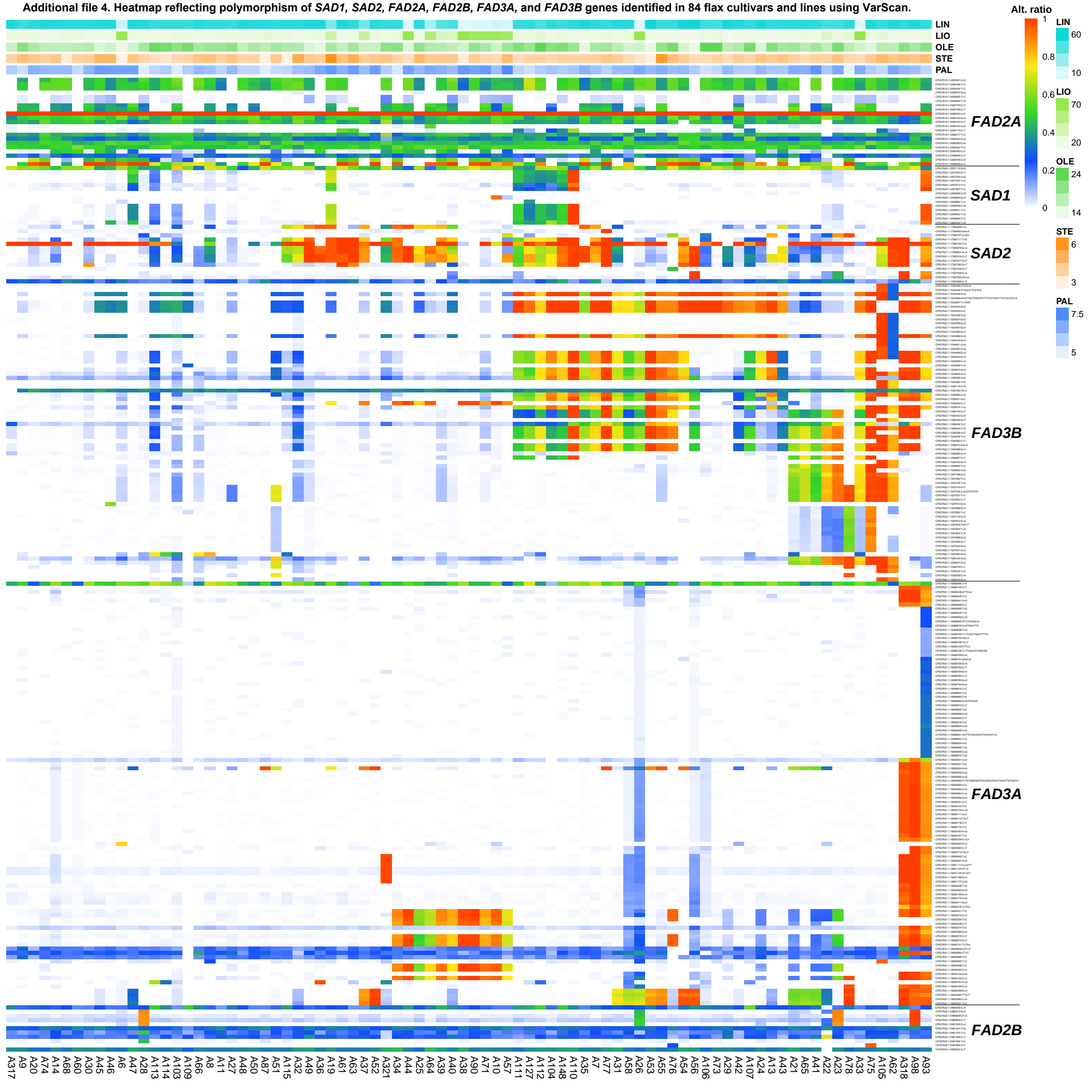

Supplement: Supplementary file 4 — Additional file 4. Heatmap reflecting polymorphisms of SAD1, SAD2, FAD2A, FAD2B, FAD3A, and FAD3B genes identified in 84 flax cultivars and lines using VarScan. Flax cultivars and lines are arranged along the horizontal axis and polymorphisms in SAD1, SAD2, FAD2A, FAD2B, FAD3A, and FAD3B genes are arranged along the vertical axis. The content of PAL (palmitic), STE (stearic), OLE (oleic), LIO (linoleic), and LIN (linolenic) and the alternative allele ratio (Alt. ratio) for each identified polymorphism are reflected in color scales for all studied cultivars and lines. [file 12870_2020_2499_MOESM4_ESM.pdf]
