# Supplementary material for: Genetic diversity of SAD and FAD genes responsible for the fatty acid composition in flax cultivars and lines
Source: BMC Plant Biol. 2020 Oct 14;20(Suppl 1):301. doi: 10.1186/s12870-020-02499-w (PMC7557025; doi:10.1186/s12870-020-02499-w)

Additional file 9.pdf. Clusterization of 84 flax cultivars and lines based on polymorphisms in *SAD1*, *SAD2*, *FAD2A*, *FAD2B*, *FAD3A*, and *FAD3B* genes revealed by freeBayes. Color scales reflect the content of fatty acids: PAL – palmitic, STE – stearic, OLE – oleic, LIO – linoleic, LIN – linolenic.

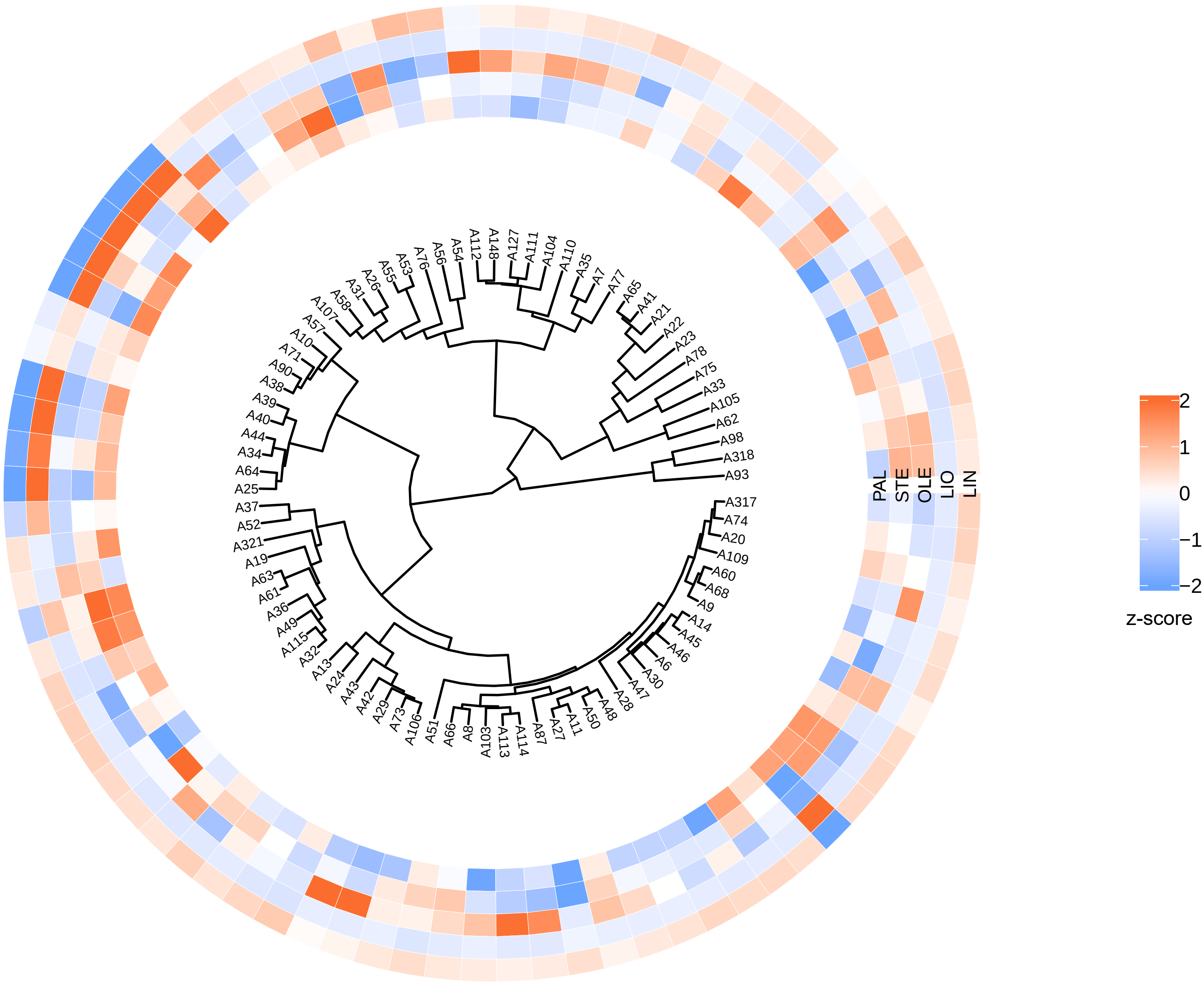

Supplement: Supplementary file 9 — Additional file 9. Clusterization of 84 flax cultivars and lines based on polymorphisms in SAD1, SAD2, FAD2A, FAD2B, FAD3A, and FAD3B genes revealed by freeBayes. [file 12870_2020_2499_MOESM9_ESM.pdf]
