# Supplementary material for: Genetic diversity of SAD and FAD genes responsible for the fatty acid composition in flax cultivars and lines
Source: BMC Plant Biol. 2020 Oct 14;20(Suppl 1):301. doi: 10.1186/s12870-020-02499-w (PMC7557025; doi:10.1186/s12870-020-02499-w)

Additional file 11. Clusterization of 84 flax samples based on polymorphisms in individual genes (*SAD1*, *SAD2*, *FAD2A*, *FAD2B*, *FAD3A*, and *FAD3B*) revealed by freeBayes. Color scales reflect the content of fatty acid: PAL – palmitic, STE – stearic, OLE – oleic, LIO – linoleic, LIN – linolenic.

SAD1 – 15 polymorphisms

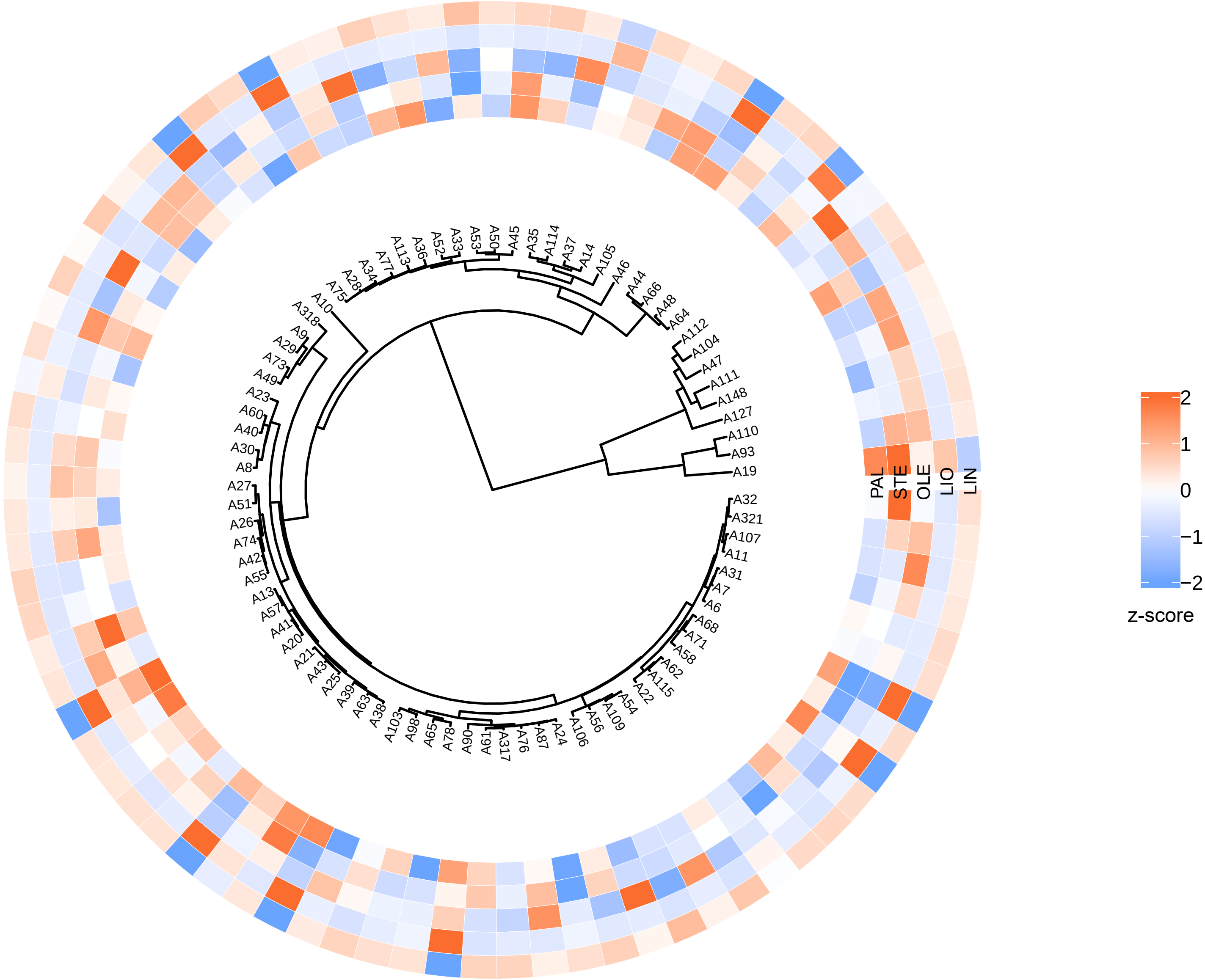

SAD2 – 15 polymorphisms

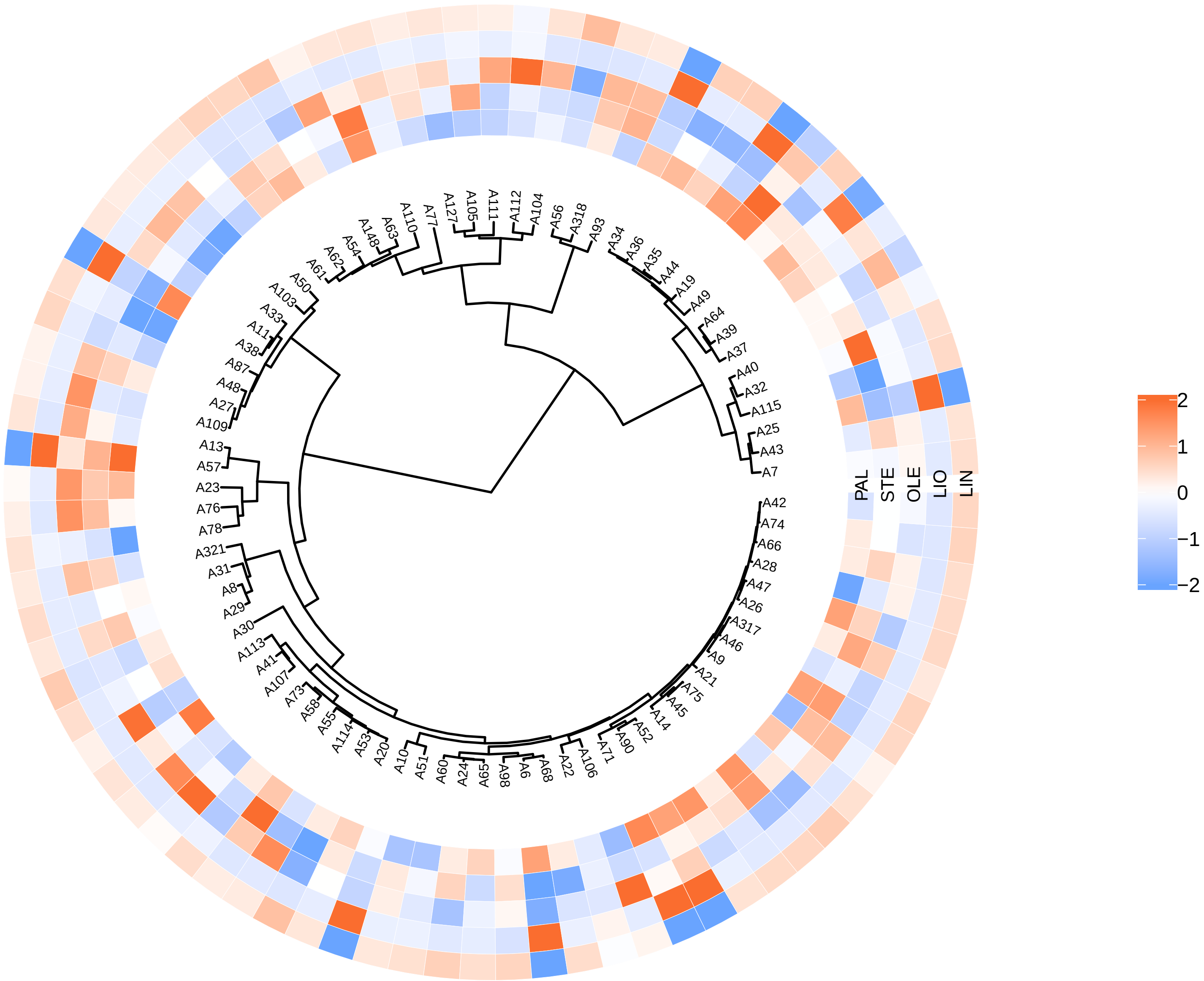

FAD2A – 31 polymorphisms

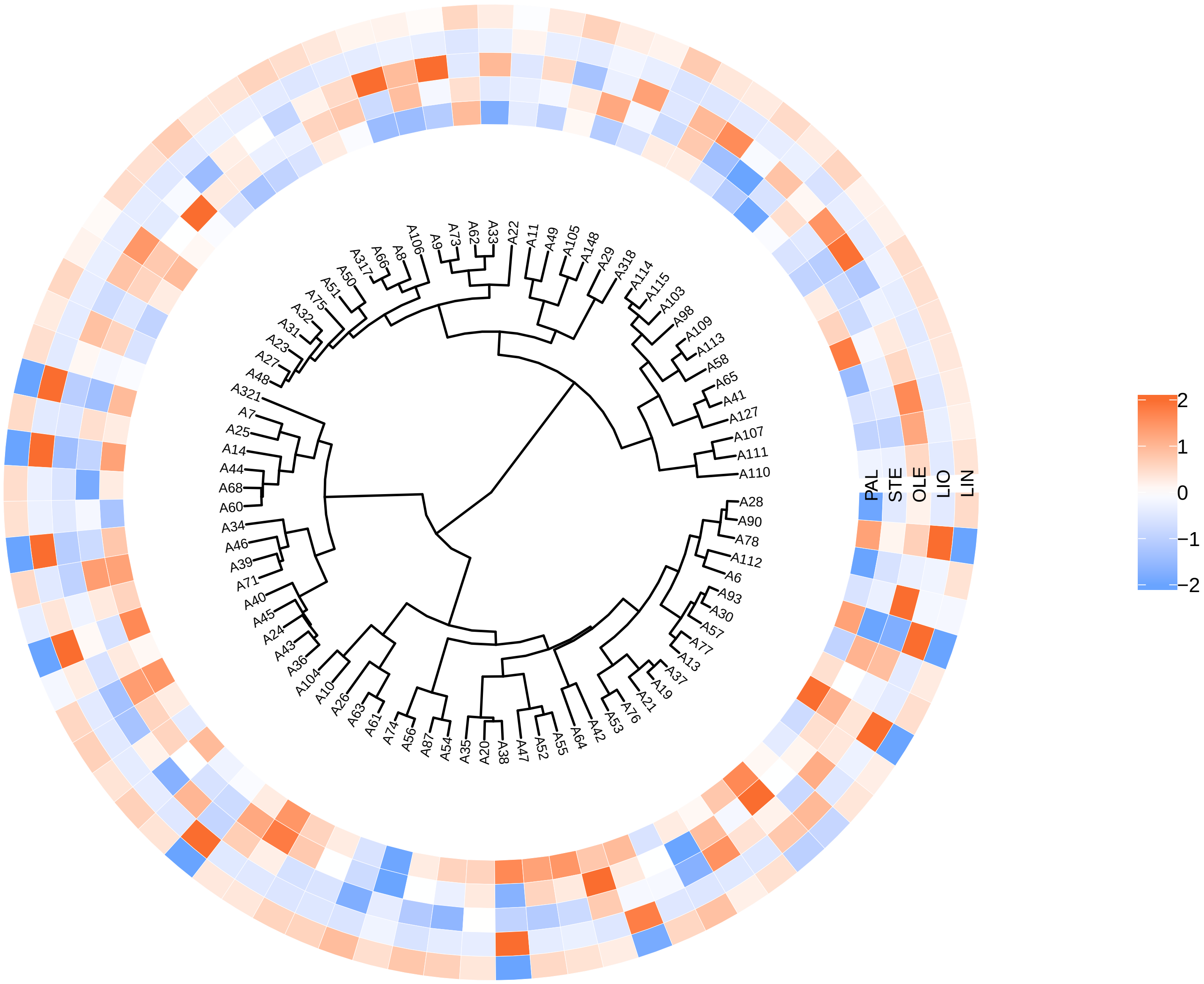

FAD2B – 15 polymorphisms

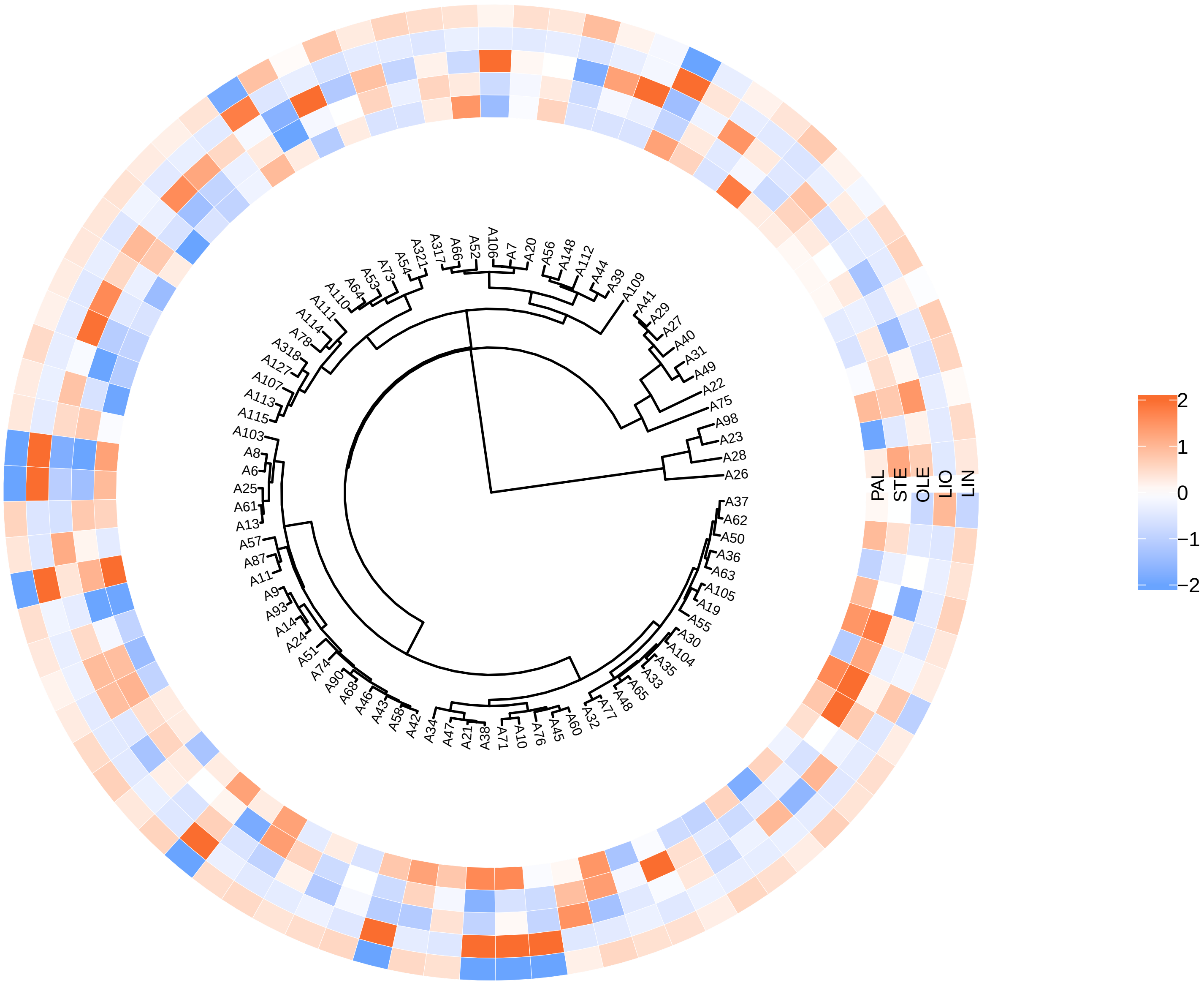

FAD3A – 90 polymorphisms

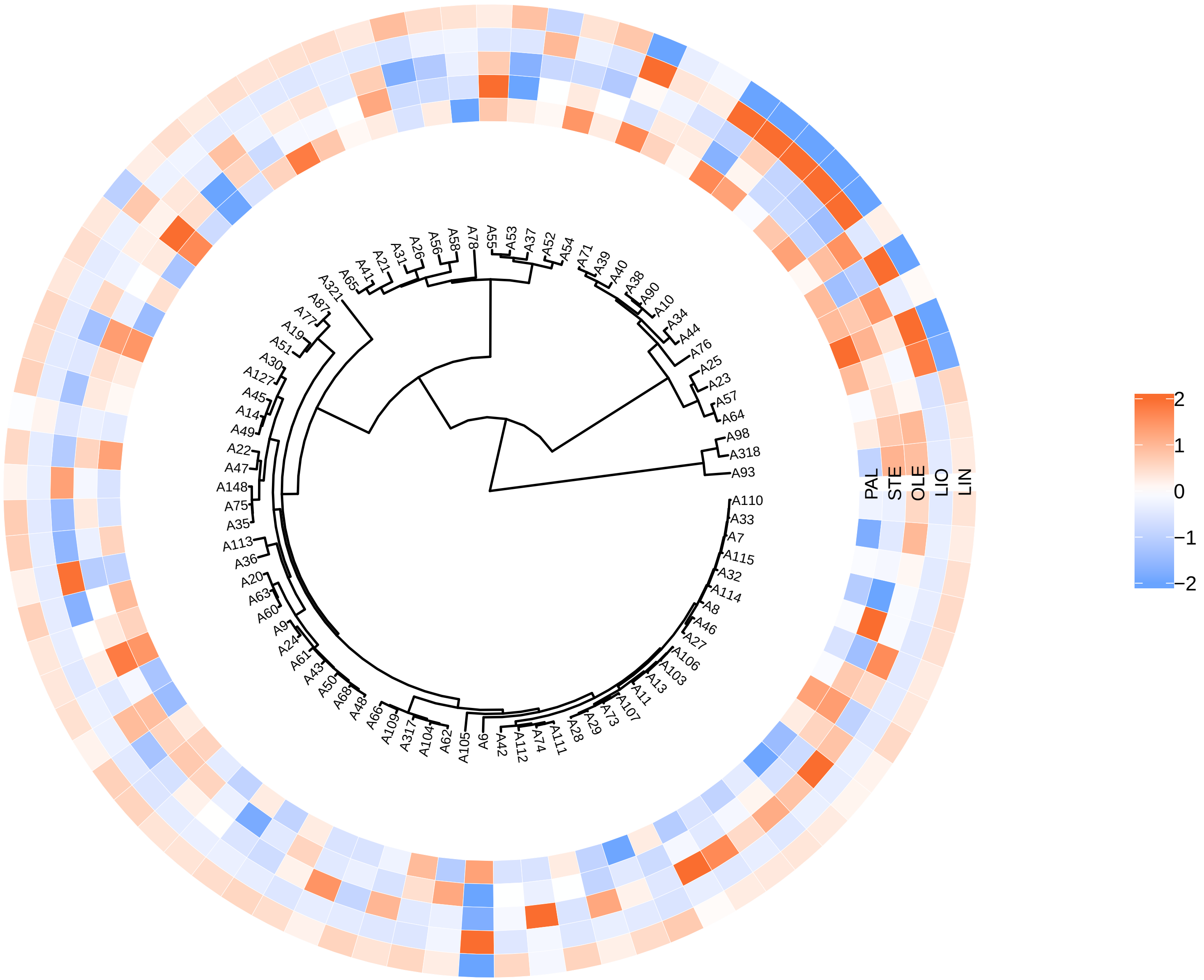

FAD3B – 73 polymorphisms

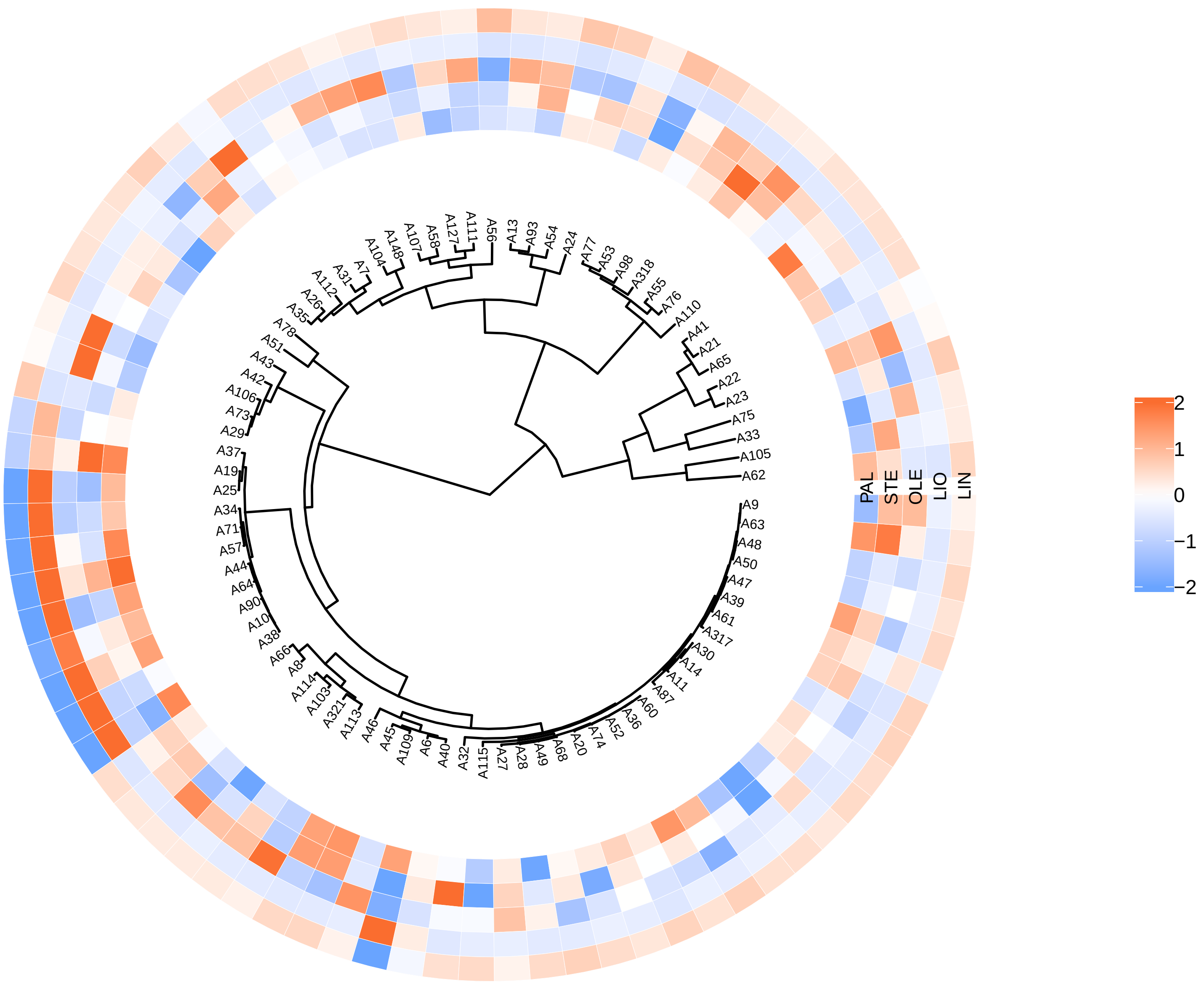

Supplement: Supplementary file 11 — Additional file 11. Clusterization of 84 flax samples based on polymorphisms in individual genes (SAD1, SAD2, FAD2A, FAD2B, FAD3A, and FAD3B) revealed by freeBayes. [file 12870_2020_2499_MOESM11_ESM.pdf]
