# Supplementary material for: Genetic diversity of SAD and FAD genes responsible for the fatty acid composition in flax cultivars and lines
Source: BMC Plant Biol. 2020 Oct 14;20(Suppl 1):301. doi: 10.1186/s12870-020-02499-w (PMC7557025; doi:10.1186/s12870-020-02499-w)
Supplement: Supplementary file 12 — Additional file 12. Primers for the first stage of DNA library preparation. [file 12870_2020_2499_MOESM12_ESM.docx]

**Additional file 12. Primers for the first stage of DNA library preparation.**

| **Primer pair** | **Sequence** | **Product length, bp** |
| --- | --- | --- |
| SAD_1_F  SAD_1_R | TCGTCGGCAGCGTCAGATGTGTATAAGAGACAGGGTCRAGTGGAGCTGAAAGC  GTCTCGTGGGCTCGGAGATGTGTATAAGAGACAGCAGCCATGAGAAAGGTGCGA | 538 |
| SAD_2_F  SAD_2_R | TCGTCGGCAGCGTCAGATGTGTATAAGAGACAGTCTCCTCCAGATCTCCTCGC  GTCTCGTGGGCTCGGAGATGTGTATAAGAGACAGACGTTSAAGGAYTGTAAGCCA | 546 |
| SAD_3_1_F  SAD_3_R | TCGTCGGCAGCGTCAGATGTGTATAAGAGACAGTCTGGGAAAATAAGTCTCTGTATCT  GTCTCGTGGGCTCGGAGATGTGTATAAGAGACAGACTTGCTCCTCGAACCCATC | 576 |
| SAD_3_2_F  SAD_3_R | TCGTCGGCAGCGTCAGATGTGTATAAGAGACAGAGCTTGTTTGAGAGAGGTTCAATA  GTCTCGTGGGCTCGGAGATGTGTATAAGAGACAGACTTGCTCCTCGAACCCATC | 539 |
| SAD_4_F  SAD_4_1_R | TCGTCGGCAGCGTCAGATGTGTATAAGAGACAGACARGATTTCCTGCCCGAA  GTCTCGTGGGCTCGGAGATGTGTATAAGAGACAGCCACTTGAACTCATTGTCAACCC | 565 |
| SAD_4_F  SAD_4_2_R | TCGTCGGCAGCGTCAGATGTGTATAAGAGACAGACARGATTTCCTGCCCGAAC  GTCTCGTGGGCTCGGAGATGTGTATAAGAGACAGTGTCAAACCTATTTGACTTCAGTGG | 557 |
| SAD_5_1_F  SAD_5_R | TCGTCGGCAGCGTCAGATGTGTATAAGAGACAGACACTGGTAGCAGCTGAAACTT  GTCTCGTGGGCTCGGAGATGTGTATAAGAGACAGTCAGAGATGCTAGGGAATCAAACA | 519 |
| SAD_5_2_F  SAD_5_R | TCGTCGGCAGCGTCAGATGTGTATAAGAGACAGACTCACATCCTATCTGCTCCT  GTCTCGTGGGCTCGGAGATGTGTATAAGAGACAGTCAGAGATGCTAGGGAATCAAACA | 576 |
| SAD_6_F  SAD_6_R | TCGTCGGCAGCGTCAGATGTGTATAAGAGACAGTGCATKTAGCAGTTTGTCCTGG  GTCTCGTGGGCTCGGAGATGTGTATAAGAGACAGGAGCTTCTCGACGATCTTGGT | 537 |
| SAD_7_F  SAD_7_R | TCGTCGGCAGCGTCAGATGTGTATAAGAGACAGGGGGACATGAAGCTGGCG  GTCTCGTGGGCTCGGAGATGTGTATAAGAGACAGTACCAATTCTCTGCTGAAGATCCA | 517 |
| SAD_8_F  SAD_8_1_R | TCGTCGGCAGCGTCAGATGTGTATAAGAGACAGGGGGAGGTGGAAAGTGGATG  GTCTCGTGGGCTCGGAGATGTGTATAAGAGACAGAGCGATTCTATCGTTGGACTGT | 500 |
| SAD_8_F  SAD_8_2_R | TCGTCGGCAGCGTCAGATGTGTATAAGAGACAGGGGGAGGTGGAAAGTGGAT  GTCTCGTGGGCTCGGAGATGTGTATAAGAGACAGAGGGATTCTATCGCTGAACTG | 497 |
| FAD2_1_F  FAD2_1_R | TCGTCGGCAGCGTCAGATGTGTATAAGAGACAGTGTTTCTGTGCTMTGAATTTTCTT  GTCTCGTGGGCTCGGAGATGTGTATAAGAGACAGACAGTTTACCGTCTTTATCAATCGT | 573 |
| FAD2_1_B_2_F  FAD2_1_B_2_R | TCGTCGGCAGCGTCAGATGTGTATAAGAGACAGTGATGAACTGTAACAGGGCCA  GTCTCGTGGGCTCGGAGATGTGTATAAGAGACAGGTTGCGTATTGAACCAGGACA | 555 |
| FAD2_2_F  FAD2_2_B_R | TCGTCGGCAGCGTCAGATGTGTATAAGAGACAGAGTCTCCCATGGTTGTMCGC  GTCTCGTGGGCTCGGAGATGTGTATAAGAGACAGGAGGTTTTGAGTAAGGAGACCG | 536 |
| FAD2_2_A_F  FAD2_2_A_R | TCGTCGGCAGCGTCAGATGTGTATAAGAGACAGTGCGTGAATGAATGAACCCAC  GTCTCGTGGGCTCGGAGATGTGTATAAGAGACAGGTGGCTTTGAGTAAGGAGACCT | 552 |
| FAD2_3_F  FAD2_3_R | TCGTCGGCAGCGTCAGATGTGTATAAGAGACAGCTTCTGCAGGTGCTGTTGAT  GTCTCGTGGGCTCGGAGATGTGTATAAGAGACAGTCTGCTTGGGGACAAAMACC | 577 |
| FAD2_3_B_2_F  FAD2_3_R | TCGTCGGCAGCGTCAGATGTGTATAAGAGACAGAAAGCAAGAATGGGTGCTGG  GTCTCGTGGGCTCGGAGATGTGTATAAGAGACAGTCTGCTTGGGGACAAAMACC | 556 |
| FAD2_4_F  FAD2_4_R | TCGTCGGCAGCGTCAGATGTGTATAAGAGACAGACTTCTCSTGGAAGCACAGC  GTCTCGTGGGCTCGGAGATGTGTATAAGAGACAGCCAGTCCCATTCGGAGGAWT | 525 |
| FAD2_5_F  FAD2_5_R | TCGTCGGCAGCGTCAGATGTGTATAAGAGACAGCACTTTCTTGCAGCACACSC  GTCTCGTGGGCTCGGAGATGTGTATAAGAGACAGRTTGTTGTACCAGAACACGCC | 404 |
| FAD2_6_F  FAD2_6_R | TCGTCGGCAGCGTCAGATGTGTATAAGAGACAGCCGCACTACAARTCCTCCGA  GTCTCGTGGGCTCGGAGATGTGTATAAGAGACAGCACAAGCCTCTGCAGCWTTG | 561 |
| FAD2_6_F  FAD2_6_A_R | TCGTCGGCAGCGTCAGATGTGTATAAGAGACAGCCGCACTACAARTCCTCCGA  GTCTCGTGGGCTCGGAGATGTGTATAAGAGACAGTGGCTCGATGGAGAAGAACAG | 565 |
| FAD3_1_F  FAD3_1_R | TCGTCGGCAGCGTCAGATGTGTATAAGAGACAGGTGGGTGGATGTTTTGTTCACC  GTCTCGTGGGCTCGGAGATGTGTATAAGAGACAGRCTCAAGTGGGCATCGGTC | 485 |
| FAD3_1_B_F  FAD3_1_B_R | TCGTCGGCAGCGTCAGATGTGTATAAGAGACAGTCAGTACGTGGGTGGATGTT  GTCTCGTGGGCTCGGAGATGTGTATAAGAGACAGAGCAAGTTCGGGCATAGGG | 515 |
| FAD3_2_F  FAD3_2_R | TCGTCGGCAGCGTCAGATGTGTATAAGAGACAGCCCCCTWGTGATCCTAAACAC  GTCTCGTGGGCTCGGAGATGTGTATAAGAGACAGGGATGCCAAGTGGATGGAGA | 558 |
| FAD3_3_F  FAD3_3_R | TCGTCGGCAGCGTCAGATGTGTATAAGAGACAGGTTGGGTGGTGTAGGCTAGT  GTCTCGTGGGCTCGGAGATGTGTATAAGAGACAGAGGAGGTCTCTCAGGACGTAG | 522 |
| FAD3_4_F  FAD3_4_R | TCGTCGGCAGCGTCAGATGTGTATAAGAGACAGTGGGTGAAGAACCCCTGGA  GTCTCGTGGGCTCGGAGATGTGTATAAGAGACAGTCTTTCTCCACATTGCCGTGA | 547 |
| FAD3_5_F  FAD3_5_R | TCGTCGGCAGCGTCAGATGTGTATAAGAGACAGAGCCACAAGACCCATCACC  GTCTCGTGGGCTCGGAGATGTGTATAAGAGACAGTCRAGAATGGGGCGGGTTG | 496 |
| FAD3_5_A_F  FAD3_5_A_R | TCGTCGGCAGCGTCAGATGTGTATAAGAGACAGCCACAAGACCCATCACCAGAA  GTCTCGTGGGCTCGGAGATGTGTATAAGAGACAGTTTTCGTCAAGAATGGGGCG | 499 |
| FAD3_6_F  FAD3_6_A_R | TCGTCGGCAGCGTCAGATGTGTATAAGAGACAGCGGACATTGTYTTGCTCGTG  GTCTCGTGGGCTCGGAGATGTGTATAAGAGACAGAAATCGTCCGGATCCATCGC | 530 |
| FAD3_6_F  FAD3_6_B_R | TCGTCGGCAGCGTCAGATGTGTATAAGAGACAGCGGACATTGTYTTGCTCGTG  GTCTCGTGGGCTCGGAGATGTGTATAAGAGACAGTCCATCGGGGTAAGACCGTA | 459 |
| FAD3_7_B_F  FAD3_7_B_R | TCGTCGGCAGCGTCAGATGTGTATAAGAGACAGGGCTACCTATTTTTAGTGTACATGC  GTCTCGTGGGCTCGGAGATGTGTATAAGAGACAGCCCTTTCTTCCCCGGACTTC | 492 |
| FAD3_7_A_F  FAD3_7_A_R | TCGTCGGCAGCGTCAGATGTGTATAAGAGACAGTTGTATGGTTTTACCCCAATGGA  GTCTCGTGGGCTCGGAGATGTGTATAAGAGACAGTTTGAATGCGAATTCCGGC | 537 |
| FAD3_8_A_F  FAD3_8_R | TCGTCGGCAGCGTCAGATGTGTATAAGAGACAGTCGTTTTCCCAGCCTTTTTCTT  GTCTCGTGGGCTCGGAGATGTGTATAAGAGACAGTGTTGATGACCCCGTAATCTC | 553 |
| FAD3_8_B_F  FAD3_8_R | TCGTCGGCAGCGTCAGATGTGTATAAGAGACAGATAGGTGTCGTTTTCCGCGT  GTCTCGTGGGCTCGGAGATGTGTATAAGAGACAGTGTTGATGACCCCGTAATCTC | 550 |
| FAD3_9_F  FAD3_9_A_R | TCGTCGGCAGCGTCAGATGTGTATAAGAGACAGCCACGGGTACGAGCAGAAG  GTCTCGTGGGCTCGGAGATGTGTATAAGAGACAGAGGSTGATCAYGTGGGTGAA | 514 |
| FAD3_9_F  FAD3_9_B_R | TCGTCGGCAGCGTCAGATGTGTATAAGAGACAGCCACGGGTACGAGCAGAAG  GTCTCGTGGGCTCGGAGATGTGTATAAGAGACAGACGACAGTGTTTACGTGGGT | 559 |
| FAD3_10_A_F  FAD3_10_R | TCGTCGGCAGCGTCAGATGTGTATAAGAGACAGACCGGAAAATGTCCTGAKCA  GTCTCGTGGGCTCGGAGATGTGTATAAGAGACAGTGGGAAAGGCCCTGATTTCT | 580 |
| FAD3_10_B_F  FAD3_10_R | TCGTCGGCAGCGTCAGATGTGTATAAGAGACAGTCCGGTTACTTTGACCTGCG  GTCTCGTGGGCTCGGAGATGTGTATAAGAGACAGTGGGAAAGGCCCTGATTTCT | 575 |
| FAD3_11_F  FAD3_11_R | TCGTCGGCAGCGTCAGATGTGTATAAGAGACAGCGTGCTGGGGAAGTACTACAG  GTCTCGTGGGCTCGGAGATGTGTATAAGAGACAGAGCAAWGCATAACTCATTTACCCAG | 571 |

*Note:* Overhang Illumina adapter sequences are marked with blue and orange; sequences that are necessary for amplification of target regions are marked with black.

**IUPAC nucleotide codes**

| **Code** | R | Y | S | W | K | M | B | D | H | V | N |
| --- | --- | --- | --- | --- | --- | --- | --- | --- | --- | --- | --- |
| **Base** | A or G | C or T | G or C | A or T | G or T | A or C | C or G or T | A or G or T | A or C or T | A or C or G | any base |
